# Consumer health informatics: enabling digital health for everyone

**DOI:** 10.29173/jchla29763

**Published:** 2024-04-01

**Authors:** Eleni Philippopoulos

**Affiliations:** Assistant Librarian, McGill University, Montreal, QC, Canada

Arnott Smith C, Keselman A. **Consumer health informatics: enabling digital health for everyone**. 1st ed. Boca Raton, FL: Chapman & Hall/CRC Press; 2020. Ebook: 239 p. ISBN: 978-0-4298-0889-0. Price: USD$47.96. Available from: https://www.routledge.com/Consumer-Health-Informatics-Enabling-Digital-Health-for-Everyone/Smith-Keselman/p/book/9780367681548

In an increasingly technology-dependant and demanding world, where more and more patients want to take charge of their health, *Consumer health information: enabling digital health for everyone* offers readers hope that patient engagement can be improved at the intersection of healthcare, information management, and information technology. It is clear, after reading this book, that information professionals need to work together with other experts in the field to adapt to the needs of consumers.

Although the book was published in December 2020, presumably the writing started before the onset of Covid-19. The authors skillfully manage to weave in the problems brought about by the pandemic into the field and offer insight as to how an ever-changing health landscape might also change the scope of consumer health informatics going forward.

The authors have published extensively in the field of consumer health and continue to grow and contribute to the field in their current roles. Catherine Arnott Smith is a professor in the Information School at the University of Wisconsin-Madison and teaches graduate level courses on digital health, information organization, and search. The topic of consumer health informatics is listed as one of her key research interests. Alla Keselman is currently with the National Library of Medicine and her published work, both leading up to and since the publication of this book, have centered on consumers and their interactions with healthcare information as well as their information seeking behaviours.

The book is divided into two main sections. The first, *Foundations*, offers readers the opportunity to get acquainted with the key terms and concepts used in consumer health informatics. Some of the terms might be too simplistic for experienced information professionals who have worked in consumer health for some time. Nevertheless, the section offers a good foundation for the reader to build their knowledge. This would be an ideal section to assign in a health informatics class in any information studies school. Non-health information professionals, such as public librarians, healthcare providers and administrators, software developers and engineers will be most informed by the concepts described and defined in the first section.

Chapter one offers readers the opportunity to get acquainted with the history of consumer health informatics, with particular attention being paid to participatory medicine, and how the internet and new technologies have contributed to empowerment of patients. Chapter two makes the important distinction between a consumer and a patient, as the terms often get conflated. It also places consumer health informatics within the larger context of biomedical, health, and medical informatics, explaining how all three come together and play an important role for the consumer.

Chapter three discusses health literacy in general, and the requirements for consumers to be able to understand their health, including general literacy problems, numeracy problems, and reading competency and comprehension issues. This information is suited for policy makers who have the ability to make concrete changes to address these problems on a wider level. However, there are also practical tips and best practices for producing consumer health information, including health organizations, medical associations, and individual healthcare professionals.

Skilled information professionals can probably skip chapters four and five, as they offer readers insight into bibliographic and citation databases as well as government and medical websites that they may already be familiar with. For less experienced librarians, or those getting started in the field, it might be a good idea to review the tools and guidelines about authority and accuracy of information laid out in chapter four.

Chapter six is useful for those who have not quite thought through how different users will interact with information. For example, there are significant differences between the way young adults and seniors interact with their health care information, what tools they are comfortable using and their levels of understanding. It is important for readers to be aware of these differences to create the best materials and tools possible.

The second part of the book, aptly entitled *Tools*, is dedicated entirely to consumer health informatic tools, which are used for personal data collection, symptom monitoring and genetic analysis, and could be used by consumers, patients and healthcare professionals. The authors discuss the potential of these tools, in particular the capabilities of patient portals in chapter seven, fitness tracking and health apps in chapter eight, and telemedicine programs and smart home features in chapters nine and ten. Those looking to develop their own tools should pay particular attention to the sections titled “Exemplar users of patient portals” and “Important app characteristics” to see examples of what kinds of apps and features meet patient needs and what users are more likely to engage positively with.

Chapter eleven is dedicated entirely to the overall ethical dilemmas that may arise with the advent of these new technologies and there are discussions specifically about each type of tools from the preceding chapters. The most discussed topic is the issue of privacy, and readers should pay particular attention to the implications laid out. One of the main concerns that is highlighted throughout the second half of the book is the potential of a breach where consumers’ personal demographic and health data are compromised. The authors stress that while these tools emerged alongside the stronghold that mobile devices have on consumers, and while they may be convenient, they come with risks. Another potential problem that was identified is the lack of an authority to oversee both the usability and the accuracy of the content presented in the apps. While it is wonderful that consumers are taking charge of their health, the chapter raise questions about the right way of doing so.

To further demonstrate these issues, Anrnott Smith and Keselman and use 23andMe as a case study. The decision is an interesting one, as it is this author’s opinion that 23andMe, although it began as a direct-to-consumer genetic testing company, has since evolved into a vanity project – the company now offers screening for more than 30 traits, including unibrow detection, sweet or salty preference, and toe length ratio [1]. The chapter read more like an in-depth review of the product and the company rather than the cautionary tale for technology developers that I assume the authors were aiming for.

One of the book’s strengths is the thorough resource list at the end of each chapter. These lists offer readers the opportunity to explore readings, websites, portals and other tools on their own. Less experienced librarians can use these sections as a starting point for compiling their own list of trusted resources to have handy when consumer health questions emerge.

Throughout the second part of the book, the authors stress the need to involve people from different fields to create the most effective tools. Overall, I believe this captures the spirit of the book in its entirety, that consumer health informatics requires that experts from different backgrounds come together for the greater good of consumers.

Given the relatively low cost of the book, I believe it is worth purchasing as it would help serve the communities discussed in this review and act as a great reference. It will be interesting to see where the field goes next, given the emergence of new technologies, and if these authors will have further insights in the future.

## References

[1] 23andMe. See our list of Personalized Genetic Reports [Internet]. 23andMe; n.d. [cited 2024 January 12]. Available from: https://www.23andme.com/en-ca/dna-reports-list/

